# Evaluation of Masticatory and Swallowing Functions Using Videoendoscopy and Observation of Facial and Mandibular Movements

**DOI:** 10.7759/cureus.95355

**Published:** 2025-10-24

**Authors:** Housei Suzuki, Masataka Watanabe, Tomoko Mukai, Tomohiro Tabata, Kazuki Ako, Mana Hirayama, Hiroyuki Suzuki, Kunihito Yamane, Yukiko Hatanaka, Junichi Furuya

**Affiliations:** 1 Oral Function Management, Showa Medical University, Graduate School of Dentistry, Tokyo, JPN

**Keywords:** dysphagia, mandibular movement, mastication, oral health, swallowing

## Abstract

Background

It is important to observe masticatory and swallowing movements to improve the eating habits of older adults with dysphagia. Observing facial and mandibular movements can be useful for such evaluations. However, the process models that occur while eating solid foods have not been adequately considered.

Objective

This study aimed to clarify the differences in facial and mandibular movements before and after bolus transport to the pharynx (stage II transport; STII) during the consumption of solid food.

Methods

As this was conducted as a pilot study, the participants included 20 healthy young adults with normal dentition who freely ate rice crackers while their facial and mandibular movements were recorded using a small mobile device. A video endoscope was inserted nasally to observe STII, and facial and mandibular movements were analyzed before (phase 1) and after (phase 2) STII. The maximum horizontal and vertical distances from the origin, velocity, total distance of movement, and the number, duration, and frequency of mastication cycles were calculated. These variables were analyzed using the Wilcoxon signed-rank test (p < 0.05).

Results

The maximum horizontal distances, maximum vertical distances, velocity, and total distances of both oral commissures and the mandible were significantly decreased in phase 2 compared with phase 1. The number, duration, and frequency of mastication also significantly decreased in phase 2 (p < 0.05).

Conclusion

Facial and mandibular movements differed between phases 1 and 2 during the consumption of solid foods. Despite the limitation that the research participants were young adults, it is important to focus on the lateral and downward movements of both oral commissures and the mandible, as well as the chewing frequency.

## Introduction

In Japan, older adults aged ≥65 years account for approximately 30% of the total population, and the number of older adults aged ≥75 years who require nursing care is rapidly increasing [1]. Among older adults residing in institutions, 51% have dysphagia [2]. Since many of the causes are related to discrepancies between swallowing ability and food texture, it is important to properly assess masticatory and swallowing functions in older adults [3]. It has been suggested that such discrepancies between masticatory and swallowing functions and food texture may be addressed through appropriate assessment [4].

Fiberoptic endoscopic evaluation of swallowing (FEES) [5] and videofluoroscopic swallowing study (VFSS) [6] are considered the gold standards for evaluating dysphagia. These tests allow visualization of food bolus transport dynamics and help determine dysphagia severity and appropriate food texture. However, frequent use of these tests is challenging due to factors such as cognitive decline among patients, and limited availability of equipment and specialized staff [7]. It is relatively common for dentists to perform FEES in Japan.

Therefore, visual observation of masticatory and swallowing movements has gained attention as a simpler and non-invasive method. Furthermore, although the present study was conducted in young adults, eating is a fundamental activity essential for health and quality of life across all ages. Thus, understanding the mechanisms of mastication and swallowing not only provides insights into age-related dysphagia but also contributes to a broader understanding of the significance of maintaining safe and efficient eating in daily life.

When eating solid food, the mandible performs both vertical and horizontal masticatory movements. To date, masticatory function has been evaluated primarily by observing mandibular movement [8,9]. As a result, the trajectory of mandibular movement with a horizontal elliptical pattern is observed in normal mastication, and the extent of horizontal movement tends to be greater in individuals with higher masticatory efficiency [10]. However, these studies did not evaluate mastication or swallowing during the actual process of eating.

A previous study reported that evaluation of the trajectory of mandibular elliptical movement was associated with dysphagia detection in patients who could chew and swallow, and that the occurrence rate of the elliptical trajectory was useful in identifying older adults who required dysphagia diet modification [11,12]. However, these studies did not consider bolus transport to the pharynx via tongue movement (stage II transport (STII)) during the consumption of solid foods. STII is a lingual squeeze-back mechanism independent of gravity, occurring intermittently during mastication to propel the bolus into the oropharynx for temporary accumulation, unlike the continuous oral propulsion of liquids that immediately triggers the pharyngeal phase. The mechanism of solid food consumption can be explained using a process model. This model is characterized by STII during food processing, in which the tongue squeezes the bolus backward, showing a different pattern of movement from that during food processing. During mastication and swallowing, the tongue, cheeks, and mandible move in coordination with each other [13]. Therefore, mandibular and facial movements may change before and after STII; however, no study has clarified this. To support eating in older adults, it may be important to understand the bolus position in the oral cavity and pharynx and the timing of STII while eating solid foods, because early penetration of the bolus into the hypopharynx could decrease the swallowing reserve in older adults [14].

Therefore, this preliminary study aimed to quantitatively analyze specific facial and mandibular movement parameters, including maximum movement distances (horizontal and vertical), velocities, and mastication cycle characteristics, before and after STII during solid food consumption in healthy young adults with natural dentition. The study sought to establish baseline kinematic data and validate the observational methodology prior to clinical application in older adults with dysphagia, where age-related changes and dental conditions may alter facial and mandibular movement patterns.

## Materials and methods

Study participants

As this study was conducted as a pilot study, the participants were young, healthy, dentate adults with no occlusal problems or decline in oral function. The study excluded individuals who could not provide consent, those in whom it was difficult to insert an endoscope because of organic abnormalities of the nasal cavity, and those whose STII was not confirmed during eating. Additionally, those who could not undergo the procedure after endoscope insertion were excluded.

None of the participants in this study met the exclusion criteria. The study contents were explained in writing and orally, and informed consent was obtained from all subjects. This study was approved by the Ethics Review Committee of Showa University (approval number: 2023-255-A).

Measurement method

**Figure 1:**
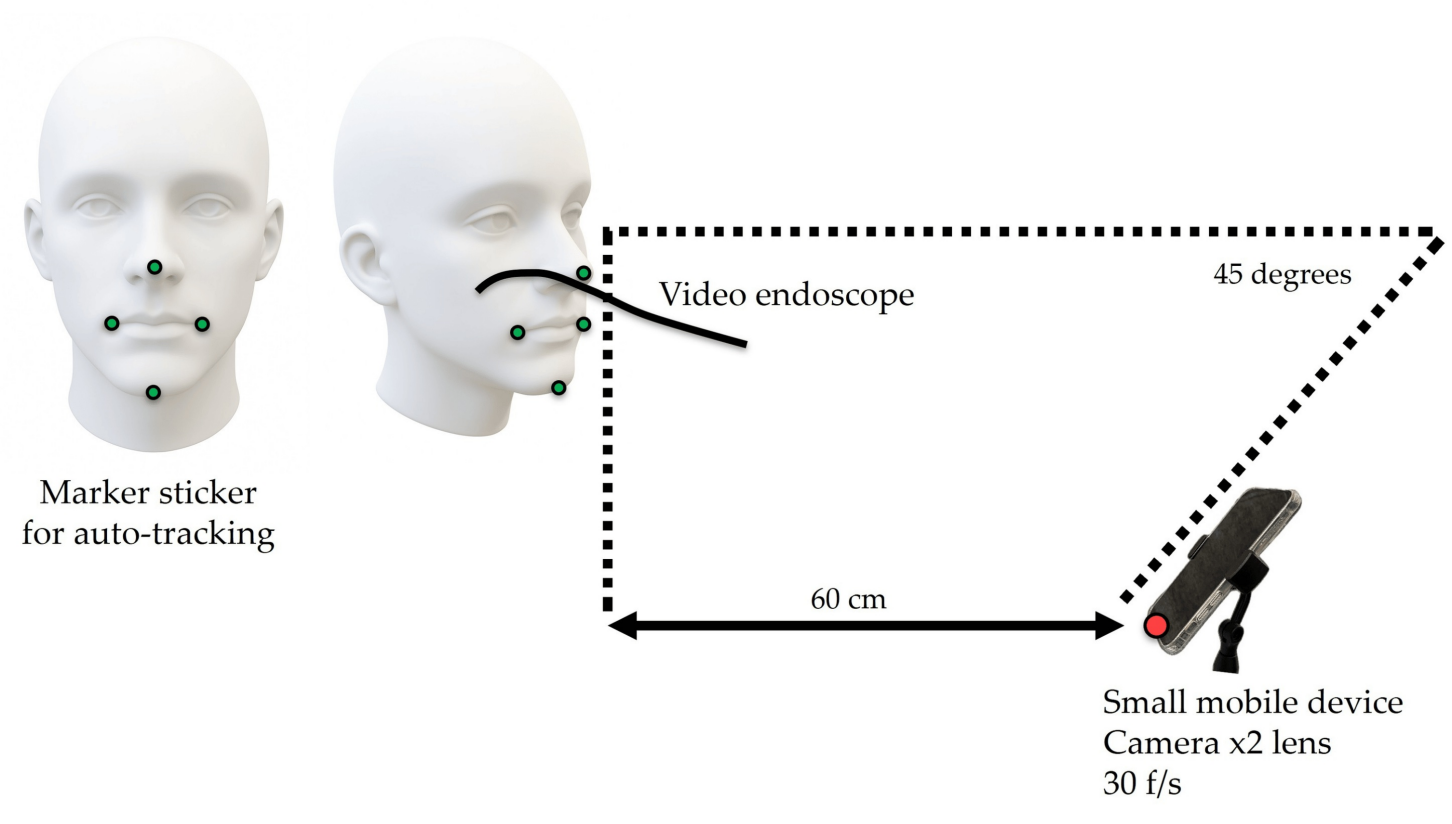
Video endoscope and mobile device settings. Marker stickers with a diameter of 5 mm were placed on the nasal apex, right and left oral commissures, and the point of maximum mandibular protrusion to track their movements, while stickers with a diameter of 3 mm were placed on top of the larger ones for more precise observation. A video endoscope was inserted nasally, and a small mobile device was fixed to the dental unit table at a 45° angle to the floor to capture facial and mandibular movements.

The participants were seated on a dental unit chair, and their heads were fixed with a headrest, as typically set for eating. For the analysis of facial and mandibular movements, black marker stickers with a diameter of 5 mm were placed on the nasal apex, right and left corners of the mouth, and the point of maximum mandibular protrusion (hereafter referred to as the "mandible"). Green stickers with a diameter of 3 mm were placed on top of these markers for easier analysis (Figure 1).

As in previous studies [11,12], 2 g of rice crackers (Happy Turn, Kameda Seika) out of 4 g was used as the test food. Participants were instructed to hold the food on their tongues while biting down with their teeth in the intercuspal position and to begin eating when given the signal. Mastication was performed without specifying the chewing side. Subsequently, the chewing side was determined from the recorded facial videos. All measurements were performed three times.

A small mobile device (iPhone 11 Pro, Apple) with a camera featuring a ×2 lens was used to capture facial and mandibular movements. The device was set up at an angle of 45° below the floor, 60 cm from the most prominent part of the mandible. The horizontal position was aligned with the midline of the face, and the height was adjusted according to each subject's sitting height so that both corners of the mouth were horizontal and the nasal apex was centered in the frame (Figure 1). A video endoscope (FNL-10RBS, PENTAX) was used to record bolus transport by STII and the whiteout during swallowing. The endoscope was inserted nasally and fixed at a high position to clearly show the uvula, root of the tongue, posterior pharyngeal wall, and epiglottis. The fiber was marked, and its position was maintained as consistently as possible during recording. The small mobile device and video endoscope recorded at a frame rate of 30 f/s, and the video data were synchronized with sound using video editing software (Adobe Premiere Pro 2024, Adobe). Furthermore, the participants were assessed using the oral hypofunction test [15].

Definition of phase

**Figure 2:**
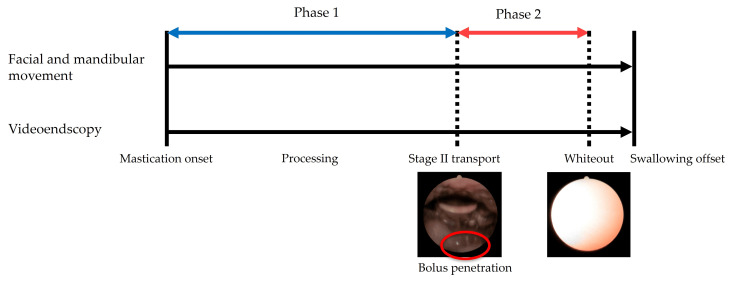
Definition of the phases during mastication and swallowing of solid foods. Phase 1 was defined as the period from the onset of mastication to just before the onset of Stage II transport, and Phase 2 was defined as the period from the onset of Stage II transport to the onset of whiteout due to swallowing. The onset of Stage II transport was defined as the time when the food bolus exceeded the palatopharyngeal arch and was visualized on the endoscopic swallowing image.

In this study, the processes of mastication and swallowing were classified into phases 1 and 2, referring to a previous study [16]. Phase 1 was defined as the period from the onset of mastication to just before the onset of STII, and phase 2 was defined as the period from the onset of STII to the onset of whiteout due to swallowing. STII onset was defined as the time when the bolus exceeded the palatopharyngeal arch and was visualized on an endoscopic swallowing image (Figure 2).

Analysis of facial and mandibular movements

**Figure 3:**
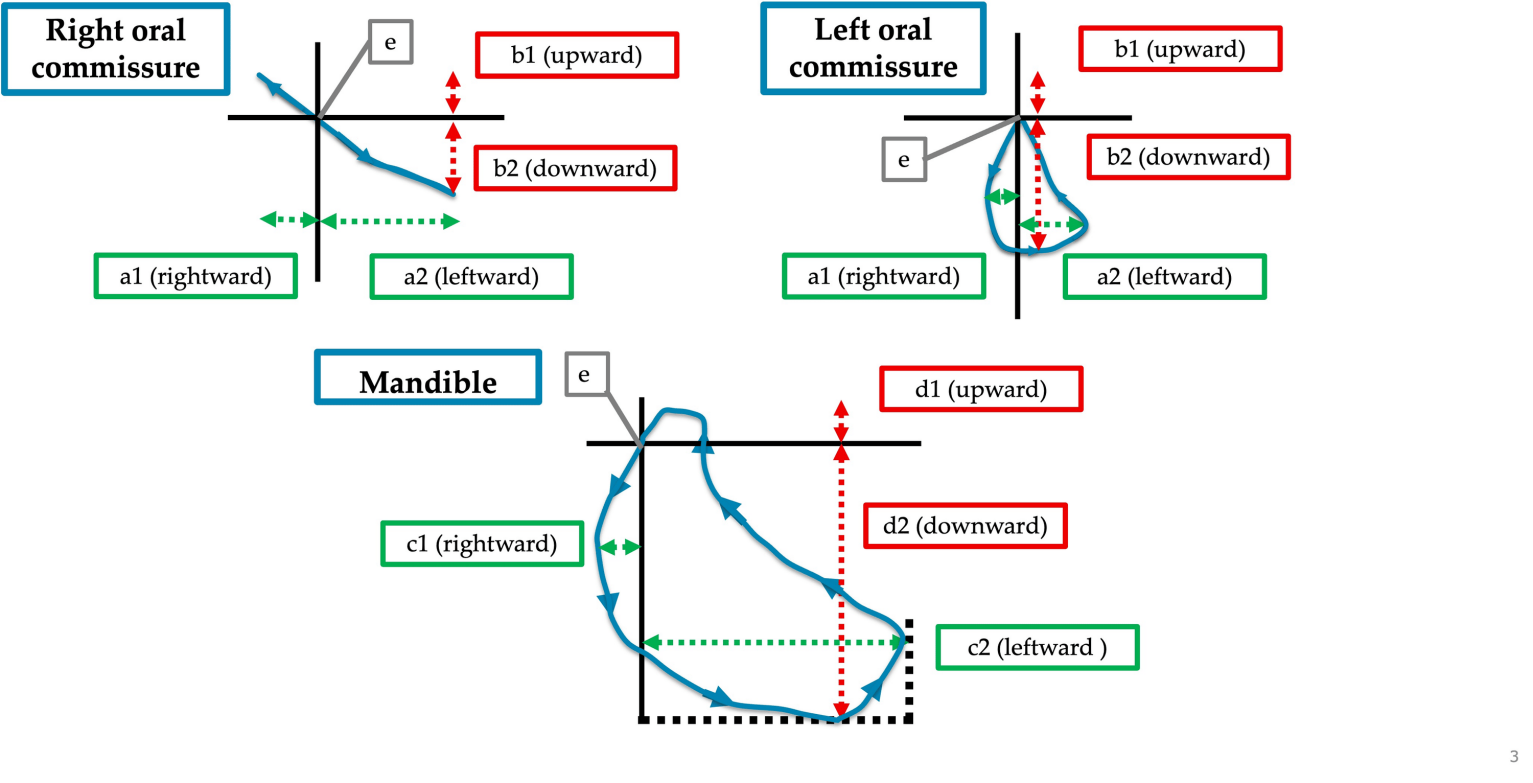
Typical movements of the oral commissures and mandible. Maximum horizontal and vertical movements of the left and right oral commissures (a, b) and mandible (c, d). The maximum distances of the oral commissures from the origin (e) were calculated in the rightward (a1), leftward (a2), upward (b1), and downward (b2) directions. The maximum distances of the mandible from the origin (e) were calculated in the rightward (c1), leftward (c2), upward (d1), and downward (d2) directions. Velocity, total movement distance, number of mastication cycles, mastication duration, and mastication frequency were also calculated. The number of mastication cycles was counted when the mandible exhibited an elliptical motion with horizontal movement from one intercuspal position to the next (e).

Synchronized videos were adjusted for head motion and origin using motion analysis software (DIPP-MOTION V, Detect), referencing the marker at the nasal apex. After performing two-dimensional calibration based on the size of the marker stickers, analysis was conducted using the automatic tracking function. This calibration allowed for the correction of errors related to the distance between the camera lens and the face. We then analyzed the movement of the left and right oral commissures and the mandible in phases 1 and 2 and calculated the maximum horizontal movement distance (leftward and rightward), maximum vertical movement distance (upward and downward), velocity, and total movement distance in each phase (Figure 3). Data from three measurements were analyzed, and the average values were used for statistical analysis.

Analysis of the mastication cycle

The number, duration, and frequency of mastication cycles in phases 1 and 2 were measured. Based on a previous study [17], one mastication cycle was defined as an elliptical motion with horizontal movement from the intercuspal position to the next intercuspal position, as shown in the video recorded by the small mobile device. Mastication frequency was calculated from the number of mastication cycles and mastication duration. Data from three measurements were analyzed, and the average values were used for statistical analysis. Two calibrated dentists performed the measurements.

Statistical method

After confirming the normality of each index (horizontal maximal movement distance, vertical maximal movement distance, mean velocity, total movement distance, number of chews, chewing time, and chewing frequency) of the oral commissures and mandible in phases 1 and 2 using the Shapiro-Wilk test, Wilcoxon’s signed-rank test was applied. Inter-rater reliability was calculated using the intraclass correlation coefficient. Statistical software (SPSS Ver. 25, IBM Japan) was used for all statistical analyses, and the significance level was set at 0.05.

## Results

The participants were 20 healthy young adults (mean age: 25.8 ± 2.2 years; 13 males and 7 females), and their mean number of teeth was 27.8 ± 0.9. The sample size was determined a priori using G*Power, with an effect size of dz = 0.7, a significance level of α = 0.05, a power (1 - β) of 0.80, and a two-tailed test, yielding a required sample size of 19. Considering potential dropouts, the final sample size was set at 20. The oral hypofunction test demonstrated a mean masticatory ability of 193.1 ± 30.7 mg/dL when assessed using gummy jelly. Habitual chewing occurred on the left side in 13 participants and on the right side in seven participants. Inter-rater reliability for mastication cycle counts was assessed using intraclass correlation coefficients, showing excellent agreement (Phase 1: ICC = 0.982, p < 0.01; Phase 2: ICC = 0.992, p < 0.01). The results of the Phase 1 and Phase 2 comparisons are shown in Table 1.

**Table 1:** Comparison of facial and mandibular movements and mastication cycles in Phases 1 and 2. Phase 1: Duration from mastication onset to Stage II transport; Phase 2: Duration from Stage II transport to whiteout. * p < 0.05, Wilcoxon signed-rank test.

Parameter	Left oral commissure			Right oral commissure			Mandible		
	Phase 1 Mean (SD)	Phase 2 Mean (SD)	p-value	Phase 1 Mean (SD)	Phase 2 Mean (SD)	p-value	Phase 1 Mean (SD)	Phase 2 Mean (SD)	p-value
Maximum leftward distance (mm)	3.2 (1.6)	2.1 (1.2)	< 0.019*	6.5 (1.9)	1.9 (1.7)	< 0.001*	7.0 (1.9)	2.8 (2.3)	< 0.001*
Maximum rightward distance (mm)	6.2 (1.3)	2.1 (1.6)	< 0.001*	2.6 (1.2)	2.4 (1.4)	0.526	6.6 (1.9)	2.7 (1.9)	< 0.001*
Maximum upward distance (mm)	3.5 (2.5)	3.0 (1.6)	0.49	2.5 (1.8)	2.7 (1.3)	0.55	3.1 (2.1)	3.9 (1.8)	0.167
Maximum downward distance (mm)	7.1 (1.4)	1.8 (1.4)	< 0.001*	6.6 (1.4)	1.4 (1.3)	< 0.001*	13.5 (3.6)	2.9 (2.6)	< 0.001*
Velocity (horizontal) (mm/s)	13.0 (4.5)	7.3 (3.1)	< 0.001*	12.6 (4.2)	7.2 (3.3)	< 0.001*	17.3 (6.0)	9.0 (4.5)	< 0.001*
Velocity (vertical) (mm/s)	17.0 (5.4)	9.3 (5.0)	< 0.001*	15.2 (5.2)	9.4 (6.4)	0.001*	27.9 (8.4)	14.1 (6.8)	< 0.001*
Total distance (horizontal) (mm)	173.5 (73.9)	19.8 (15.1)	< 0.001*	163.8 (70.6)	18.4 (11.8)	< 0.001*	229.8 (98.4)	31.0 (39.6)	< 0.001*
Total distance (vertical) (mm)	237.9 (103.8)	23.5 (19.0)	< 0.001*	242.9 (185.5)	21.6 (18.9)	< 0.001*	365.6 (130.7)	34.1 (27.0)	< 0.001*
Number of mastication cycles (times)							18.0 (6.5)	1.4 (1.4)	< 0.001*
Duration (s)							13.5 (4.3)	1.9 (1.2)	< 0.001*
Frequency (times/s)							1.4 (0.3)	0.6 (0.5)	< 0.001*

**Figure 4:**
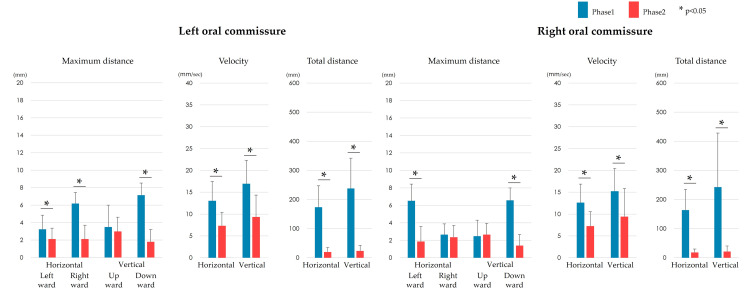
Comparison of facial movements between Phases 1 and 2. The maximum upward distance showed no significant difference between the phases. In contrast, the leftward horizontal distance, maximum downward distance, mean velocity, and total movement distance of the left and right oral commissures were significantly lower in Phase 2 than in Phase 1 (p < 0.05).

**Figure 5:**
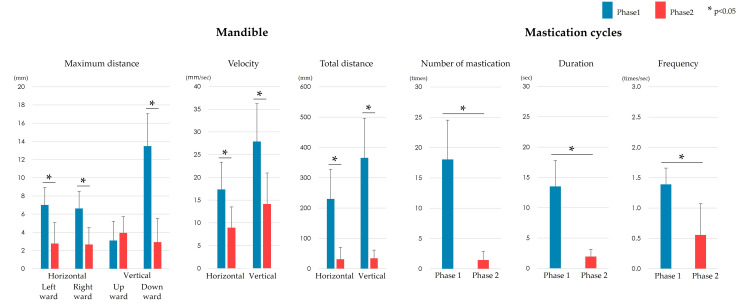
Comparison of mandibular movements and mastication cycles between Phases 1 and 2. The movement of the mandible showed no significant difference between Phases 1 and 2 in terms of the maximum upward distance. In contrast, the maximum leftward and rightward distances, maximum downward distance, mean velocity, and total movement distance were significantly lower in Phase 2 than in Phase 1 (p < 0.05). One mastication cycle was defined as an elliptical motion with horizontal movement from one intercuspal position to the next. The number of mastication cycles, mastication duration, and mastication frequency decreased significantly (p < 0.05) in Phase 2 compared with Phase 1.

The movements of the left and right oral commissures and mandible showed almost the same trend. In particular, the maximum distance of downward movement showed the largest difference between the phases (Figures 4-5). However, the maximum distance of rightward movement of the left oral commissure showed a significant difference between the phases, whereas that of the right oral commissure did not. The distance of upward movement at all measurement points showed no significant differences between the phases. The mean velocity, total movement distance, number of mastication cycles, mastication duration, and mastication frequency were significantly lower in Phase 2 than in Phase 1.

## Discussion

This study aimed to elucidate the changes in facial and mandibular movements during the mastication and swallowing of solid food. The results showed that facial and mandibular movements changed significantly before and after bolus transport to the pharynx, and that the horizontal and downward movement distances became shorter after bolus transport. The frequency of elliptical trajectories, including horizontal movements of the mandible, which indicate mastication cycles, decreased after bolus transport. These results suggest that it is possible to determine the timing of bolus transport to the pharynx and the position of the bolus through facial and mandibular observation without using direct methods such as VESS.

In this study, we observed the movements of the right and left oral commissures and the mandible in young dentate adults. Therefore, it was assumed that both the left and right oral commissures would show similar movement trends. However, there was a difference in the maximum distance of rightward movement, which was thought to be due to the habitual chewing side. In this study, habitual chewing occurred on the left side in most participants (13 of 20), suggesting that mastication mainly occurred on the left side. During mastication, the cheek plays a role in retrieving escaped food boluses from the buccal vestibule and repositioning them on the occlusal surfaces. Consequently, the maximum distance of rightward movement in Phase 1 could be larger in the left commissure than in the right commissure due to this compensatory role.

In Phase 2, the maximum distance of downward movement, velocity, and total movement distance in both the oral commissures and mandible were significantly reduced compared with the findings in Phase 1. A previous study that evaluated masticatory movements using electromyography of the suprahyoid muscles suggested that, as mastication progressed, the activity of the suprahyoid muscles and the mastication cycle decreased [18]. Mastication involves vertical and lateral movements of the mandible, and the magnitude and speed of these movements are thought to decrease as food is crushed and the bolus is formed. In a previous study that compared tongue and mandibular movements during mastication using fluoroscopy [19], the vertical movements of the mandible were larger during mastication and smaller during STII and swallowing. These previous studies support the results of the present study.

The decrease in maximum distance, velocity, and total movement distance after STII was attributed to changes in tongue movement, as the cheeks, tongue, and mandible are functionally linked during mastication and swallowing. A previous study reported that tongue movement changes during the transition from oral processing of the food bolus to its transport to the pharynx [20]. Mastication is a coordinated movement involving the cheek, tongue, and mandible. At the beginning of STII, the tongue performs a squeezing motion known as “squeeze back” and shifts to a different movement pattern from that seen during mastication, thereby affecting the movements of the oral commissures and mandible.

As a result of changes in mandibular movements, the number of mastication cycles, mastication duration, and mastication frequency, which indicate oral motor changes, decreased significantly after STII, as previously reported [16]. In this study, the number of mastication cycles was counted when an elliptical trajectory of the mandible with horizontal movement was observed. It was considered that the frequency of mastication decreased because the hardness of the food diminished as mastication progressed, and STII occurred intermittently when the food was sufficiently chewed and mixed with saliva to form an appropriate bolus. One study reported that sensory inputs from oral mechanoreceptors, muscle spindles within the masticatory muscles, and periodontal mechanoreceptors modulate the basic rhythmic output of the central pattern generator, thereby adjusting the masticatory force, velocity, and trajectory [21]. Similarly, previous studies showed that mastication duration decreases as mastication progresses and that the mandibular movement pathway significantly decreases with the progression of mastication [22,23]. These previous findings support the results of the present study.

In the present study, there were no significant differences in the maximum distance of upward movement at the left and right oral commissures and mandible between Phases 1 and 2. Upward movement from the intercuspal position is considered limited during mastication, particularly in dentate individuals without tooth loss, resulting in a relatively short vertical displacement. Therefore, it was considered that the maximum upward movement distance at both the oral commissures and mandible was not a key factor in observing facial and mandibular movements during the mastication and swallowing of solid foods.

In this study, 2 g of typical Japanese rice crackers was used as the test food, and participants were instructed to eat freely to observe natural chewing and swallowing [24]. The chewing side was not restricted, as participants were expected to eat in a manner consistent with their daily habits. A previous study reported that changes in the chewing side affect the rhythm of mastication but not masticatory function [25]. The number of STIIs in masticatory swallowing is reportedly higher when a specific chewing frequency or duration is not indicated [24]. Based on these findings, this study employed free chewing to reproduce the participants’ natural mastication and swallowing behaviors.

This study had several limitations. First, as it was conducted only in healthy young participants with natural dentition and normal oral function, the actual facial and mandibular movements of older adults remain unknown. Their movement patterns may differ from those of younger individuals owing to age-related muscle weakness and changes in coordination [26]. Second, the test food consisted solely of rice crackers, which is also a limitation. The dynamics of mastication and swallowing change significantly depending on the physical properties of foods [27]. It is therefore necessary to verify these results using foods with different textures, such as semi-solid foods. Previous studies have reported that mastication frequency and food processing differ according to food texture [28]. Third, in this study, facial and mandibular movements were evaluated only in two dimensions. It has been reported that the oral commissures are pulled in a posterior centrifugal direction by contraction of the buccal muscles during mastication [29]. Therefore, three-dimensional motion analysis is necessary to accurately capture muscle activity during such complex movements. Fourth, regarding the FEES assessment, the discomfort caused by this procedure is unavoidable and may have influenced the participants’ natural eating behaviors during the examination.

However, the observational method used in this study can be easily applied in many clinical settings. The results of this study appear useful for the clinical observation of eating behaviors in older adults and suggest that specific observable characteristics of facial and mandibular movements may help detect STII and determine bolus position during solid food intake.

## Conclusions

The movements of the oral commissures and mandible during mastication and swallowing change before and after bolus transport from the oral cavity to the pharynx via the tongue. In particular, it is important to focus on the range and speed of the lateral and downward movements of the oral commissures and mandible.
